# Crystal structure, equation of state, and elasticity of phase H (MgSiO_4_H_2_) at Earth’s lower mantle pressures

**DOI:** 10.1038/srep15534

**Published:** 2015-10-23

**Authors:** Jun Tsuchiya, Mainak Mookherjee

**Affiliations:** 1 Geodynamics Research Center, Ehime University, 2–5 Bunkyo-cho, Matsuyama, Ehime 790–8577 JAPAN; 2 Earth-Life Science Institute Ehime Satellite, 2–5 Bunkyo-cho, Matsuyama, Ehime 790-8577 JAPAN; 3 Earth and Atmospheric Sciences, Cornell University, 2122 Snee Hall, Ithaca, NY 14853 USA; 4 Earth, Ocean, and Atmospheric Sciences, Florida State University, Tallahassee, FL 32306 USA

## Abstract

Dense hydrous magnesium silicate (DHMS) phases play a crucial role in transporting water in to the Earth’s interior. A newly discovered DHMS, phase H (MgSiO_4_H_2_), is stable at Earth’s lower mantle, i.e., at pressures greater than 30 GPa. Here we report the crystal structure and elasticity of phase H and its evolution upon compression. Using first principles simulations, we have explored the relative energetics of the candidate crystal structures with ordered and disordered configurations of magnesium and silicon atoms in the octahedral sites. At conditions relevant to Earth’s lower mantle, it is likely that phase H is able to incorporate a significant amount of aluminum, which may enhance the thermodynamic stability of phase H. The sound wave velocities of phase H are ~2–4% smaller than those of isostructural δ-AlOOH. The shear wave impedance contrast due to the transformation of phase D to a mixture of phase H and stishovite at pressures relevant to the upper part of the lower mantle could partly explain the geophysical observations. The calculated elastic wave velocities and anisotropies indicate that phase H can be a source of significant seismic anisotropy in the lower mantle.

Water plays a crucial role in sustaining geological activity of our planet by affecting melting relationships^1^ in the solid Earth and also influencing its rheology^2^,^3^,^4^. Hence, considerable research has been conducted to understand the transport of water into the Earth’s interior^5^ and the thermodynamic stability^6^,^7^ and the elasticity^8^ of hydrous mineral phases. It is known that ocean water interacts with the underlying oceanic crusts and the exposed peridotite along related bending faults in the oceanic crusts^9^,^10^ and forms hydrous minerals such as serpentine. As the oceanic plates subduct, the hydrous phases, including serpentine, are dragged along with the plate, and upon compression serpentine transforms to DHMS phases^11^,^12^. A series of high-pressure experimental studies have been performed to understand the thermodynamic stability of DHMS phases. These experiments demonstrated that phase D (MgSi_2_O_6_H_2_) was thermodynamically stable at the Earth’s lower mantle conditions and did not show any further transformation to any other denser hydrous phase^13^,^14^, whereas there have been suggestions that phase D might decompose or transform above 44 GPa^15^,^16^.

Recently, first principles simulations predicted a new DHMS phase, phase H (MgSiO_4_H_2_)^17^, to be stable at pressures greater than the stability field of phase D. First principles simulations predicted that upon compression (~40 GPa) phase D should decompose to phase H and a high-pressure polymorph of silica (SiO_2_), stishovite. This prediction was later confirmed by high-pressure experiments using *in situ* X-ray diffraction measurements with the sintered diamond multi-anvil apparatus^18^. Phase H has a crystal structure consisting of magnesium (Mg) and silicon (Si) atoms in an octahedral coordination and is similar to the crystal structure of δ-AlOOH^17^,^18^ where aluminum (Al) atoms occur in octahedral sites. Owing to the possibility of the configurational disorder of the Mg and Si atoms in the octahedral sites, the space group symmetry of phase H was subsequently refined using the single-crystal X-ray diffraction method^19^.

Despite being an important candidate for transporting water into the deep Earth, the crystal structure and elasticity of phase H at high pressures are unknown. In this study, using first principles simulations, we report the crystal structure of phase H including the effect of ordering and disordering of Mg and Si atoms in the octahedral sites. We also determine the equation of state, full-elastic constant tensor, and the elastic anisotropy of phase H at pressures relevant to Earth’s lower mantle pressure conditions.

## Results

**Figure 1:**
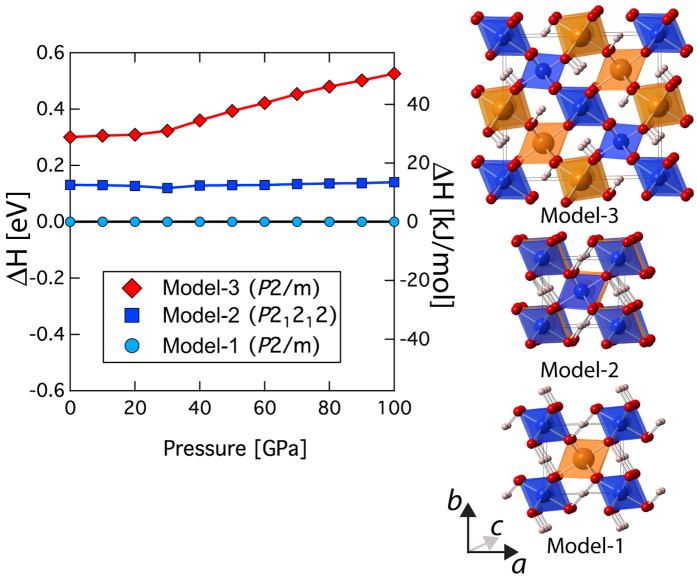
Energetics of order-disorder in phase H. Left panel shows the plot of enthalpy as a function of pressure for model-1, model-2, and model-3. In the right panel, the optimized zero pressure crystal structures for model-1, model-2, and model-3 are shown.

**Figure 2:**
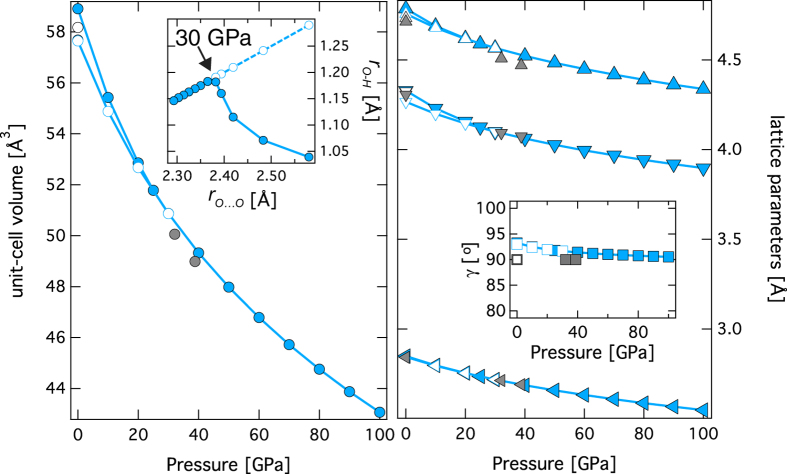
Pressure dependence of unit-cell volume and lattice parameters. The left panel shows the plot of unit-cell volume as a function of pressure for phase H with ordered Mg and Si atoms (model-1) (light blue filled symbol). Also, shown are the metastable extensions of unit-cell volume for the symmetric hydrogen bonded structure (light blue open symbols). Inset shows the plot of *r*_*O−H*_ as a function of 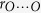, at around 30 GPa, 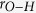 becomes (1/2) of 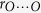, i.e., hydrogen bond symmetrizes. The right panel shows the plot of the ***a***-, ***b***-, and ***c***-axes as a function of pressure. Inset shows the plot of γ as a function of pressure. For comparison, the experimental results are also plotted (open symbols)^18^ and (grey filled symbols)^19^.

Energetics calculations, considering the effects of Mg and Si order-disorder reveal that the model-1 with *P*2/m space group symmetry has the lowest enthalpy across all pressures relevant for Earth’s interior (Fig. 1). Model-2 with space group symmetry of *P2*_*1*_*2*_*1*_*2* show slightly greater enthalpy (~0.13 eV/Z, where Z = formula unit in the unit-cell) than model-1. The pressure-volume results for phase H are well described by the finite strain formulation of Birch-Murnaghan^20^ (Fig. 2) with a zero pressure bulk modulus *K*_*O*_ of 147.5 (±6.8) GPa, the pressure derivative of the zero pressure bulk modulus 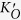 of 4.9 (±0.2), and a zero pressure unit-cell volume *V*_*O*_ of 58.9 (±0.2) Å^3^. The predicted lattice parameters are in good agreement with the experimental results^18^,^19^ (Fig. 2, Table 1, Supplementary Table 1). The predicted monoclinic distortion in γ, i.e., deviation from orthogonal angle (90°) is minor and remains within ±3° at all pressures explored in this study. Upon compression, the hydroxyl bond (*r*_*O−H*_) increases in its length and the distance between the pair of oxygen atoms 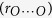 decreases, ultimately forming a symmetric hydrogen bond, i.e., 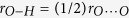 at ~30 GPa. The hydrogen bond symmetrization has also been predicted for an isostructural dense hydrous phase such as δ-AlOOH phase using first principles simulations^21^,^22^ and later confirmed by experiments^23^,^24^.

**Figure 3:**
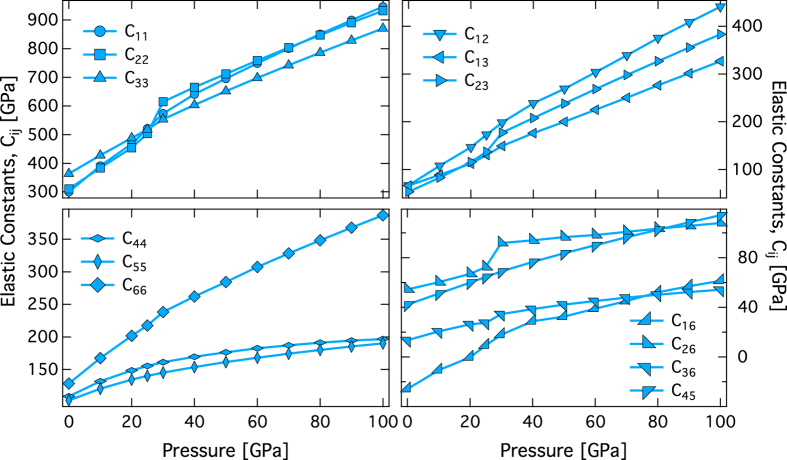
Elastic constants as a function of pressure. Plot shows the full elastic constant tensor for phase H with ordered Mg and Si atoms (model-1) as a function of pressure, upper left panel shows the principle elastic constants- C_11_, C_22_, and C_33_; upper right panel shows the off diagonal elastic constants- C_12_, C_13_, and C_23_; lower left panel shows the shear elastic constants- C_44_, C_55_, and C_66_; and the lower right panel shows the off diagonal elastic constants- C_16_, C_26_, C_36_, and C_45_. The discontinuous behavior of C_11_, C_22_, C_23_, C_16_, C_26_, C_66_ is related to the hydrogen bond symmetrization at 30 GPa.

The components of the full elastic constant tensor of phase H increase with pressure (Table 2, Fig. 3). The principal elastic constants C_11_ and C_22_, the off-diagonal elastic constants C_23_, C_16_, and C_26_, and the shear elastic constant C_66_ show anomalous increase at ~30 GPa (Fig. 3). The pressure dependent anomalous behavior in the elastic constant is predicted for both the ordered (model-1) and disordered (model-2) phase H structures (Supplementary Table 2). The likely cause for such pressure dependent anomalous behavior in elasticity is the symmetrization of hydrogen bonds. Similar anomalous behavior in elasticity for the isostructural δ-AlOOH phase have also been explained by hydrogen bond symmetrization^21^. In the present study, although, the anomalous behavior occurs at ~30 GPa, it is possible that the quantum and thermal vibrational effects may affect the pressure where hydrogen bond symmetrizes and also the onset of anomalous behaviors^25^. At pressures beyond the hydrogen bond symmetrization, all the elastic constants stiffen steadily upon compression without further pressure dependent anomalous behavior. It is interesting to note that the predicted C_33_ is smaller than C_11_ and C_22_ at pressures beyond the hydrogen bond symmetrization (i.e., 30–100 GPa), though the crystal structure of phase H consists of edge shearing SiO_6_ and MgO_6_ octahedral units which are densely packed along the *c*-axis. Since the Mg-O and Si-O bonds are not directly aligned along the *c*-axis, compression along the *c*-axis can be achieved relatively easily by the alteration of the Si-O-Si and Mg-O-Mg angles. The elastic constants C_11_ and C_22_ of the model-2 structure are similar to those of model-1, whereas C_33_ of model-2 is about 5% smaller than that of model-1 (Supplementary Table 2). The model-2 type phase H is likely to be mechanically unstable at low pressure conditions since the shear elastic constant, C_55_ of <0 at ~0 GPa.

**Figure 4:**
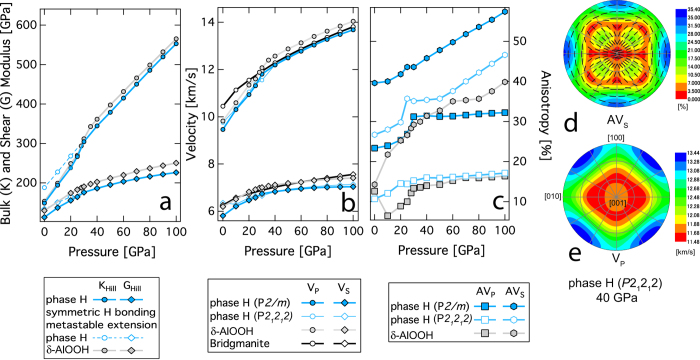
Pressure dependence of elasticity and anisotropy. (**a**) Plot of bulk and shear moduli as a function of pressure, (**b**) plot of primary (V_P_) and shear (V_S_) velocity as a function of pressure, (**c**) plot of shear wave polarization anisotropy (AV_S_) and primary wave azimuthal anisotropy (AV_P_) as a function of pressure. Stereographic projection for (**d**) AV_S_ and (**e**) V_P_ for phase H (model-2) at 40 GPa, clearly indicates orthorhombic symmetry.

The bulk (*K*), shear (*G*) moduli, primary (V_P_), and shear (V_S_) velocities for phase H increase upon compression (Fig. 4). The bulk sound velocity (i.e., V_Φ_ = (K/ρ)^0.5^) is in very good agreement with the recent study on shock wave experimental studies on DHMS phases^26^ (Supplementary Fig. 1). The discontinuous behavior of *K* at pressures of ~20–30 GPa is related to the anomalous increase of the principal elastic constants, C_11_ and C_22_, which is in turn related to the hydrogen bond symmetrization. The effect of Mg-Si order disorder do not show appreciable changes in the bulk and shear moduli throughout the range of pressures explored in this study (Fig. 4). Since phase H and δ-AlOOH are isostructural at conditions relevant to the lower mantle, these two phases could show appreciable solid solution through Mg + Si = 2Al substitutions. This has been observed in recent experimental studies^18^,^27^. We notice that in comparison to the aluminous end member δ-AlOOH phase, the primary (V_P_), and shear (V_S_) velocities are 3% and 4% slower for phase H (Fig. 4), respectively. However, the primary (V_P_) sound velocity for the volumetrically dominant lower mantle phase, bridgmanite, lies in between that of δ-AlOOH and phase H. The shear sound velocity (V_S_) for bridgmanite lies in between that of δ-AlOOH and phase H for a pressure range of 30 to 60 GPa but is greater than both δ-AlOOH and phase H above 60 GPa (Fig. 4). Hence, lower velocities in the deep mantle could be due to a combination of iron enrichment, higher temperatures, and the presence of deeply subducted hydrous phases.

## Discussions

The sound wave velocities vary as a function of the propagation direction. The anisotropy in the sound wave velocities could be calculated by solving the Christoffel’s equations, det|*c*_*ijkl*_*n*_*j*_*n*_*l*_-ρ*V*^2^δ_ik_| = 0, where ***n***, ρ, *V*, and δ_ik_ are the propagation direction, density, wave velocity, and Kronecker delta, respectively^28^. The calculated shear wave (V_S_) polarization anisotropy (AV_S_) is defined as AV_S_ = 100 × (V_S1_–V_S2_)/V_S_ and the azimuthal anisotropy for primary wave (AV_P_) is defined as A_P_ = 100 × (V_Pmax_–V_Pmin_)/V_P_. The polarization anisotropy AV_S_ increases with pressure, whereas the azimuthal anisotropy AV_P_ remains unchanged beyond the hydrogen bond symmetrization pressure ~30 GPa. The azimuthal anisotropy AV_P_ is reduced for the model-2 type disordered structured phase H (AV_P_ ~ 18%) compared with that of model-1 (AV_P_ ~ 32%) (Fig. 4, Supplementary Figs 2 and 3), it is still higher than that of bridgmanite (AV_P_ ~ 12%)^29^, and post perovskite (AV_P_ ~ 15%)^30^ at lower mantle pressures of 100 GPa.

There is no major change of the fast and slow directions of the velocities of phase H between 40 and 100 GPa. The core mantle boundary region is known to be seismically anisotropic and the horizontally polarized shear waves (V_SH_) propagate faster than vertically polarized ones (V_SV_)^31^. The calculated polarization anisotropy indicates that if the lattice preferred orientation of phase H is developed by aligning the *c*-axis vertically, the high polarization anisotropy with V_SH_ > V_SV_ could partly explain the observed seismic anisotropy at the bottom of lower mantle.

**Figure 5:**
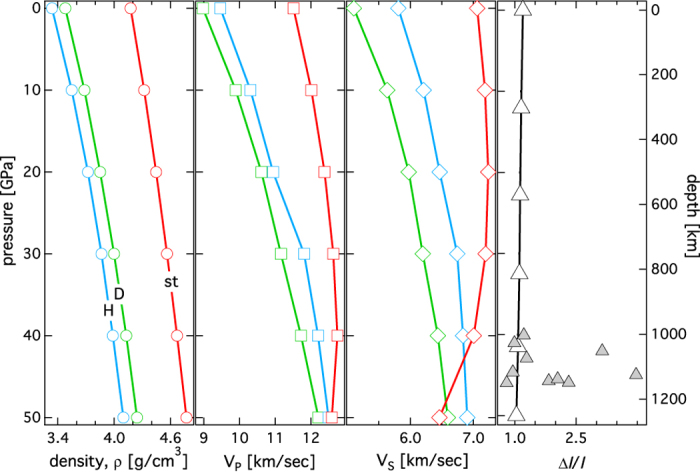
Density, velocity, and impedance contrast. Plot of (**a**) density, (**b**) primary wave velocity (V_P_), and (**c**) shear wave velocity (V_S_) vs. pressure for phase H (model-1) (H: light blue open symbols), phase D (D: light green open symbol), and stishovite (st: red open symbols). (**d**) plot of impedance contrast (ΔI/I) (open triangles) defined as the sum of shear velocity contrast and density contrast^44^ across the reaction phase D = phase H+ stishovite. Also shown are the depth dependent impedance contrasts from seismological observations (filled grey triangles)^31^.

We also estimated the acoustic impedance contrast (ΔI/I) due to the decomposition of phase D to a mixture of phase H and stishovite at pressure conditions relevant for the upper part of the lower mantle (Fig. 5). Decomposition reaction involving the DHMS phases could partly explain observed seismic impedance contrast at a mid-mantle depths of ~1000 km^32^. If the impedance contrast is indeed partly related to the decomposition of phase D to phase H and stishovite, it would mean that a significant amount of water could be transported into the deep Earth through DHMS phases such as phase D and phase H down to lower mantle depths.

## Methods

We used first principles calculations based on the density functional theory to predict the structure, equation of state, and elasticity of phase H. We used generalized gradient approximation (GGA)^33^ for the description of exchange-correlation functional. Norm-conserving pseudopotentials^34^ have been employed to describe the ionic core potentials of silicon (Si), oxygen (O), and hydrogen (H), whereas the magnesium (Mg) pseudopotential is generated by the method of U. von Barth and R. Car^35^. The semi-core p-electrons are not included in the Mg psedopotential. These potentials were extensively tested in previous studies^35^,^36^,^37^. GGA has been successfully used in predicting high-pressure behavior of hydrous phases^38^,^39^ and have been tested experimentally^40^. All structural parameters are fully relaxed at a static 0 K and 0–100 GPa by the damped variable cell shape molecular dynamics method implemented in the Quantum-Espresso codes^41^ until residual forces become less than 1.0 × 10^−5^ Ry/au. The electronic wave function is expanded in plane waves using a kinetic energy cutoff of 80 Ry. The irreducible Brillouin zone of the phase H structure is sampled on a 4 × 4 × 6 Monkhorst-Pack mesh^42^. In addition to the unit cell calculation of phase H, we also conducted the supercells in order to estimate the effect of disordering between Mg and Si onto the cell parameters. K-points in those supercells are sampled on a larger mesh in order to achieve the k-point sampling, which is equivalent to that for the unit cell in reciprocal space. The elastic constants are determined by using the stress-strain relations^43^. The magnitude of all applied strains was ±1%. The linear relation was ensured for this strain range.

Recent first principles simulations reported a fully optimized crystal structure of phase H^17^ with a monoclinic symmetry (γ ~ 91° at 40 GPa) with space group *P*2/m that was slightly distorted from orthorhombic symmetry. Experimental studies reported that the structure of phase H is orthorhombic with space group *P*2_1_nm^18^ and more recently based on single-crystal X-ray diffraction, a space group of *P*nnm^19^ (CaCl_2_ type structure) has been proposed. Although, the polyhedral frameworks of crystal structures proposed by all these studies are similar, the Mg and Si were found to be disordered in the octahedral sites^19^. In order to mimic the effect of disorder of the Mg and Si in the octahedral sites, we used distinct structural models and evaluate their relative energetics (Fig. 1). Figure 1 shows the calculated model crystal structures. All these models are derivative of the previous first principles simulations with *P*2/m^17^ space group symmetry. Model-1 corresponds to the ordered Mg and Si crystal structure (cell size ***a*** × ***b*** × ***c***)^17^, Model-2 (***a*** × ***b*** × 2***c*** supercell) where Mg and Si atoms are alternatively arranged along the *c*-axis resulting in an orthorhombic space group symmetry *P*2_1_2_1_2, and Model-3 (2***a*** × 2***b*** × ***c*** supercell) where Mg and Si atoms are alternatively placed along the ***a***- and ***b***- axes with monoclinic space group symmetry *P*2/m (Fig. 1). We have fully optimized the cell parameters of these model structures at 0–100 GPa. We also calculate the full elastic constant tensor for model-1 and model-2 (Supplementary Table 1). In order to compare the elasticity of phase H with that of the major lower mantle phase, we have also calculated the elastic constants of bridgmanite using the same pseudopotentials as those used for the calculation of phase H (Supplementary Fig. 1).

## Additional Information

**How to cite this article**: Tsuchiya, J. and Mookherjee, M. Crystal structure, equation of state, and elasticity of phase H (MgSiO_4_H_2_) at Earth's lower mantle pressures. *Sci. Rep.* **5**, 15534; doi: 10.1038/srep15534 (2015).

## Supplementary Material

Supplementary Information

## Figures and Tables

**Table 1:** Cell parameters, hydroxyl (*r*_*O−H*_) bond distances, oxygen-oxygen 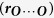 bond distances, and polyhedral volumes of phase H (model-1) as a function of pressure.

P	ρ	*a*	*b*	*c*	γ	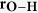	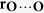	MgO_6_	SiO_6_
(GPa)	(g/cm^3^)	(Å)	(Å)	(Å)	(°)	(Å)	(Å)	(Å^3^)	(Å^3^)
0	3.338	4.786	4.328	2.848	93.3	1.039	2.580	12.1	8.1
10	3.547	4.689	4.230	2.797	92.5	1.071	2.483	11.3	7.8
20	3.720	4.620	4.157	2.754	92.0	1.115	2.419	10.7	7.5
25	3.797	4.590	4.126	2.735	91.8	1.160	2.394	10.4	7.4
30	3.865	4.567	4.100	2.718	91.7	1.182	2.382	10.2	7.3
40	3.986	4.523	4.062	2.686	91.4	1.183	2.365	9.8	7.2
50	4.098	4.484	4.027	2.658	91.2	1.175	2.350	9.5	7.0
60	4.202	4.449	3.996	2.632	91.0	1.169	2.338	9.2	6.9
70	4.300	4.418	3.968	2.609	90.9	1.163	2.325	9.0	6.7
80	4.393	4.389	3.943	2.587	90.7	1.157	2.314	8.7	6.6
90	4.481	4.362	3.919	2.567	90.6	1.152	2.303	8.5	6.5
100	4.565	4.338	3.897	2.548	90.5	1.146	2.293	8.4	6.4

**Table 2:** Elastic constants (C_ij_), bulk (K) and shear (G) moduli of phase H (model-1) as a function of pressure.

P	ρ	C_11_	C_12_	C_13_	C_22_	C_23_	C_33_	C_16_	C_26_	C_36_	C_44_	C_55_	C_66_	C_45_	K_Hill_	G_Hill_
(GPa)	(g/cm^3^)	(GPa)	(GPa)	(GPa)	(GPa)	(GPa)	(GPa)	(GPa)	(GPa)	(GPa)	(GPa)	(GPa)	(GPa)	(GPa)	(GPa)	(GPa)
0	3.338	300.9	66.8	66.5	311.0	53.3	364.1	−25.8	54.4	13.6	108.8	102.5	128.4	42.1	148.7	112.7
10	3.547	391.1	108.0	88.8	384.0	83.0	428.1	−10.6	60.2	20.6	131.8	120.4	167.3	51.0	194.0	136.9
20	3.720	468.1	146.6	112.3	454.6	115.8	488.5	−0.3	67.0	26.3	148.6	134.4	201.6	59.9	237.6	155.5
25	3.797	520.9	172.9	130.5	504.1	137.0	517.9	9.2	72.3	27.4	155.5	140.3	217.6	64.4	266.7	164.5
30	3.865	573.3	198.2	149.4	614.6	177.2	553.4	17.9	91.8	34.5	161.1	145.2	238.4	68.8	304.4	175.8
40	3.986	641.1	238.6	176.2	665.2	207.7	603.8	28.8	93.9	38.6	169.4	153.6	262.1	76.5	344.5	186.2
50	4.098	695.3	269.0	200.1	712.0	238.1	651.6	32.5	96.5	42.1	176.4	161.3	284.4	83.5	379.0	195.2
60	4.202	749.0	303.8	225.3	758.4	268.5	697.7	38.9	98.2	45.0	182.6	168.1	307.4	90.0	415.0	203.4
70	4.300	800.8	338.9	250.4	803.3	297.8	742.2	45.1	100.8	47.7	187.2	174.3	328.3	96.4	450.3	210.2
80	4.393	850.2	375.1	276.2	847.2	326.7	785.8	52.1	103.3	50.2	191.2	180.0	348.4	102.4	485.7	216.2
90	4.481	898.8	408.2	301.5	890.3	355.3	828.6	57.0	105.6	52.3	194.3	185.3	367.9	108.4	519.6	221.6
100	4.565	946.1	440.9	326.6	932.1	383.2	870.2	61.4	107.8	54.2	196.7	190.2	386.9	114.1	552.9	226.3
