# Supplementary material for: Crystal structure, equation of state, and elasticity of phase H (MgSiO4H2) at Earth’s lower mantle pressures
Source: Sci Rep. 2015 Oct 23;5:15534. doi: 10.1038/srep15534 (PMC4616034; doi:10.1038/srep15534)
Supplement: Supplementary Information [file srep15534-s1.pdf]

Supplementary Information

Crystal structure, equation of state, and elasticity of phase H ($MgSiO_4H_2$) at Earth's lower mantle pressures

Jun Tsuchiya[1,2*] and Mainak Mookherjee[3]

[1]Geodynamics Research Center, Ehime University, 2-5 Bunkyo-cho, Matsuyama, Ehime 790-8577 JAPAN.

[2]Earth-Life Science Institute, Ehime Satellite, 2-5 Bunkyo-cho, Matsuyama, Ehime 790-8577 JAPAN.

[3]Earth and Atmospheric Sciences, Cornell University, 2122 Snee Hall, Ithaca, NY 14853 USA.

*corresponding author: Jun Tsuchiya (junt@ehime-u.ac.jp)

Supplementary Table 1. Cell parameters, hydroxyl (r_{O-H}) bond distances, oxygen-oxygen ($r_{O\cdots O}$) bond distances, and polyhedral volumes of phase H (model-2) as a function of pressure. Note that model-2 has orthorhombic symmetry ($P2_12_12$) above ~40 GPa.

P (GPa)	ρ (g/cm^3)	***a*** (Å)	***b*** (Å)	***c*** (Å)	β (°)	r_{O-H} (Å)	$r_{O\cdots O}$ (Å)	MgO_6 (Å^3)	SiO_6 (Å^3)
0	3.339	4.721	4.234	5.893	89.20	1.028, 1.051	2.537, 2.517	11.445,11.440	8.365 8.363
10	3.546	4.633	4.146	5.774	89.61	1.055, 1.086	2.456, 2.442	10.741,10.746	7.980, 7.979
20	3.716	4.566	4.082	5.679	89.77	1.088, 1.135	2.401, 2.391	10.222,10.225	7.694, 7.694
30	3.856	4.513	4.036	5.599	90.00	1.122, 1.162	2.368, 2.367	9.804, 9.806	7.467, 7.467
40	3.980	4.468	3.999	5.530	90.00	1.170, 1.173	2.346, 2.351	9.456, 9.458	7.277, 7.278
50	4.092	4.430	3.968	5.468	90.00	1.166, 1.169	2.333, 2.338	9.157, 9.158	7.111, 7.111
60	4.196	4.395	3.940	5.412	90.00	1.161, 1.163	2.321, 2.326	8.894, 8.895	6.962, 6.963
70	4.294	4.365	3.914	5.360	90.00	1.156, 1.157	2.310, 2.314	8.661, 8.662	6.828, 6.828
80	4.387	4.336	3.890	5.313	90.00	1.150, 1.152	2.299, 2.304	8.452, 8.452	6.705, 6.705
90	4.476	4.310	3.868	5.270	90.00	1.146, 1.147	2.289, 2.294	8.263, 8.263	6.592, 6.592
100	4.560	4.286	3.848	5.229	90.00	1.141, 1.142	2.280, 2.284	8.090, 8.090	6.487, 6.487

Supplementary Table 2. Elastic constants (Cij), bulk (K_{Hill}) and shear (G_{Hill}) moduli of phase H (model-2) as a function of pressure.

**P** (GPa)	ρ (g/cm^3)	C_{11}	C_{12}	C_{13}	C_{22}	C_{23}	C_{33} (GPa)	C_{44}	C_{55}	C_{66}	K_{Hill}	G_{Hill}
0	3.339	254.3	64.2	105.3	286.8	10.5	303.0	93.1	-79.3	142.8	137.4	135.0
10	3.546	394.9	93.5	104.0	407.0	72.7	390.9	116.5	108.9	183.7	192.4	140.4
20	3.716	480.5	134.7	127.7	489.1	110.6	450.9	130.5	124.0	214.5	240.4	160.2
25	3.790	519.6	160.8	142.7	541.2	135.2	481.5	134.1	127.4	228.7	268.3	167.5
30	3.856	548.2	174.5	154.4	567.4	152.1	508.0	137.9	119.3	242.5	286.8	170.6
35	3.921	594.8	211.9	172.2	619.7	176.9	537.6	140.7	129.2	257.8	318.2	179.1
40	3.980	635.3	243.0	189.3	661.6	197.9	562.1	142.9	136.3	271.8	344.8	185.9
50	4.092	692.3	277.4	213.9	709.9	228.1	610.1	147.6	146.4	292.4	381.5	196.6
60	4.196	746.4	311.1	239.3	755.7	258.2	655.4	151.1	152.2	312.2	417.3	205.2
70	4.294	798.9	344.8	264.9	800.3	288.1	699.3	153.6	157.3	331.6	452.6	212.9
80	4.387	849.5	378.2	290.7	843.9	317.8	742.1	155.5	161.6	350.3	487.4	220.0
90	4.476	898.7	411.1	316.6	886.9	347.2	783.9	156.9	165.4	368.7	521.7	226.5
100	4.560	946.7	443.8	342.5	929.1	376.2	824.7	157.8	168.6	386.7	555.6	232.6

Supplementary Figure 1. The calculated velocities of phase H, δ-AlOOH, and $MgSiO_3$ bridgmanite are compared with experiments. In order to compare the elasticity of phase H with that of the major lower mantle phase, we have calculated the elastic constants of $MgSiO_3$-Bridgmanite (Mg-Br). We used generalized gradient approximation (GGA) and the same norm-conserving pseudopotentials as those used for the calculation of phase H. The irreducible Brillouin zone of Mg-Br is sampled on a 4 × 4 × 2 Monkhorst-Pack mesh. As already discussed in the previous study[43], the velocities based on GGA are generally a few percent lower than experiments. The blue and red open squares are experimental velocities of Mg-Br at 300 K by ultrasonic measurements[44]. Bulk sound velocity of phase H (green open squares) is estimated from shock experiments[25,45].

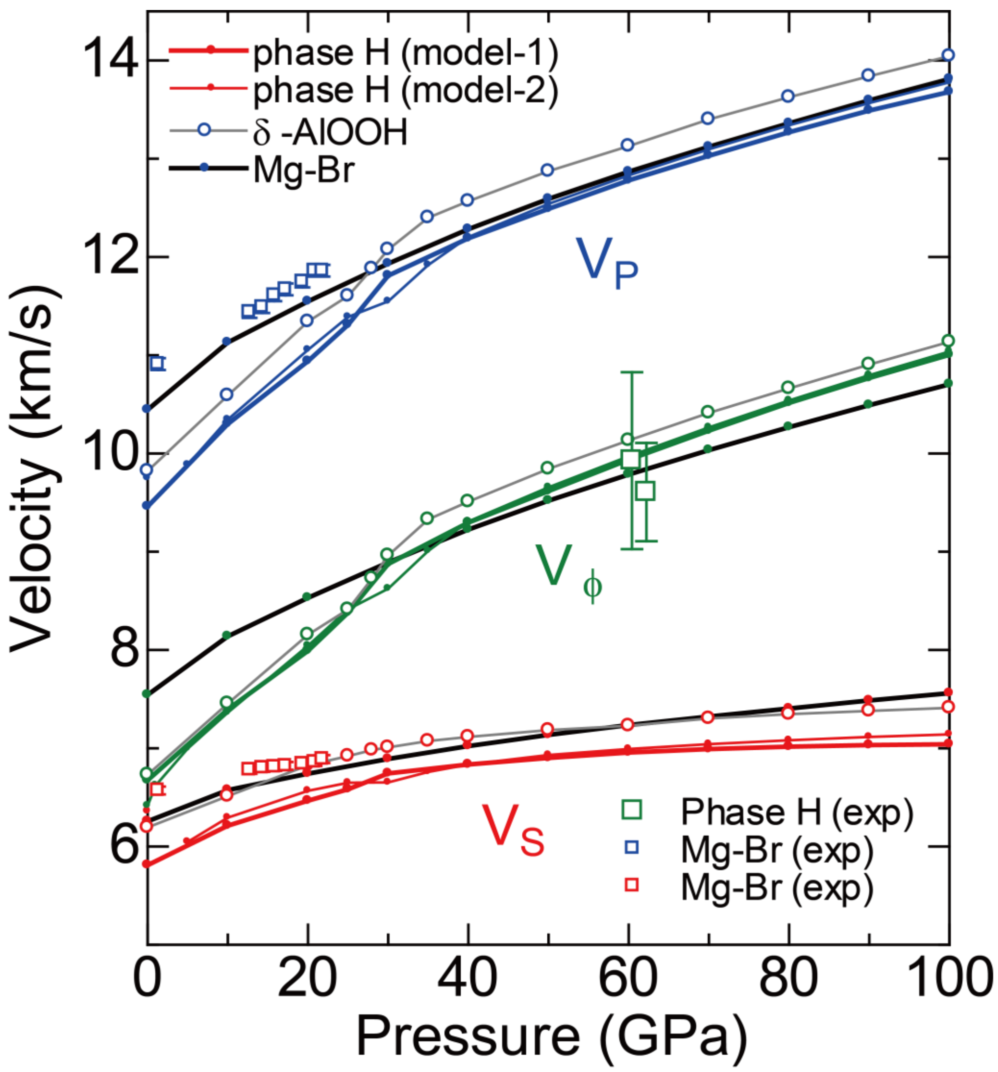

Supplementary Figure 2. Elastic wave velocities (V_P, V_{S1}, and V_{S2}) , V_P/V_{S1} velocity ratio, and polarization anisotropy (AV_S) of phase H (model-1 and model-2) at 40 GPa. Grid lines are drawn at every 30 degrees.

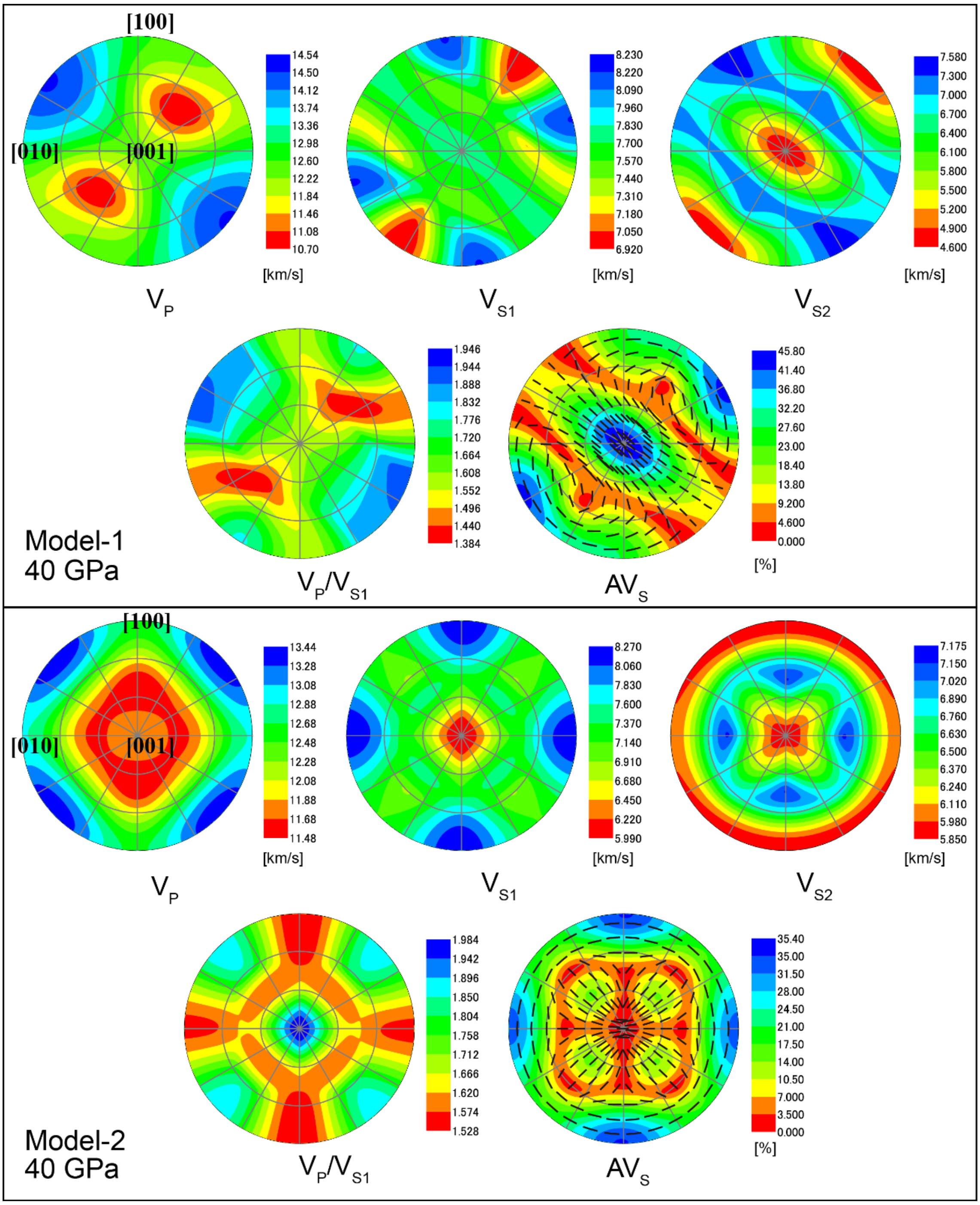

Supplementary Figure 3. Elastic wave velocities (V_P, V_{S1}, and V_{S2}) , V_P/V_{S1} velocity ratio, and polarization anisotropy (AV_S) of phase H (model-1 and model-2) at 100 GPa. Grid lines are drown at every 30 degrees.

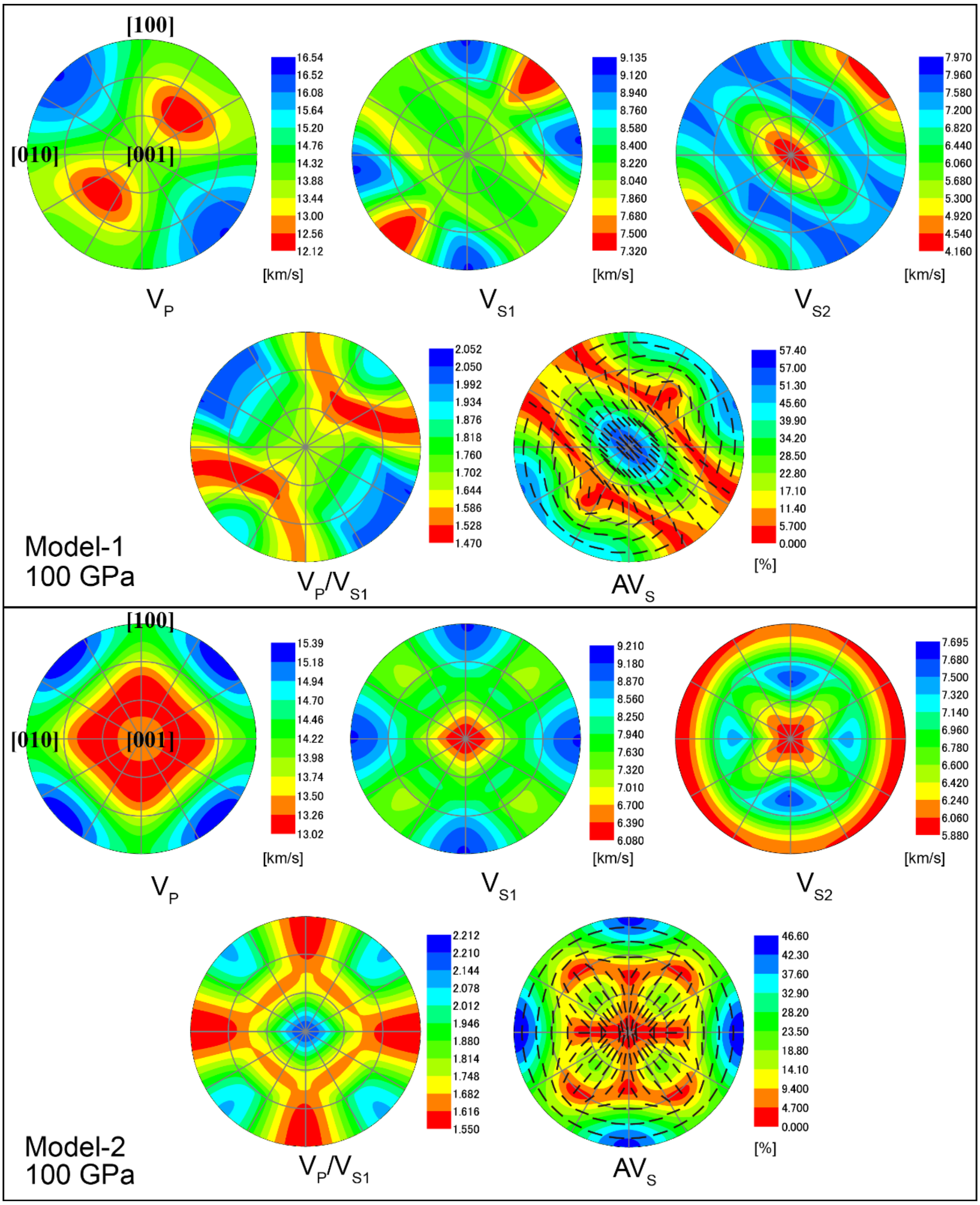

References

43. Mookherjee, M. and J. Tsuchiya, Elasticity of superhydrous phase B, $Mg_{10}Si_3O_{14}(OH)_4$, *Phys. Earth Planet. Int.* **238**, 42-50, doi:10.1016/j.pepi.2014.10.010 (2015)

44. Chantel, J., D.J. Frost, C.A. McCammon, Z. Jing, Y. Wang, Acoustic velocities of pure and iron-bearing magnesium silicate perovskite measured to 25 GPa and 1200 K, *Geophys. Res. Lett.* **45**. L19307, doi:10.1029/2012GL053075 (2012).

45. Zhang, Y., T. Sekine, Y. Yu, H. He, C. Meng, F. Liu, M. Zhang, Hugoniot and sound velocity of antigorite and evidence for sluggish decomposition, *Phys. Chem. Minerals.* **41**, 313-322, doi:10.1007/s00269-013-0650-0 (2014).
